# Based on the Geometric Characteristics of Binocular Imaging for Yarn Remaining Detection

**DOI:** 10.3390/s25020339

**Published:** 2025-01-09

**Authors:** Ke Le, Yanhong Yuan

**Affiliations:** 1 School of Mechanical Engineering, Zhejiang Sci-Tech University, Hangzhou 310018, China; le.cc@foxmail.com; 2 Zhejiang Provincial Innovation Center of Advanced Textile Technology, Shaoxing 312000, China; 3 Key Laboratory of Modern Textile Machinery Technology of Zhejiang Province, Hangzhou 310018, China

**Keywords:** yarn margin detection, binocular vision, rotary body, imaging model

## Abstract

The automated detection of yarn margins is crucial for ensuring the continuity and quality of production in textile workshops. Traditional methods rely on workers visually inspecting the yarn margin to determine the timing of replacement; these methods fail to provide real-time data and cannot meet the precise scheduling requirements of modern production. The complex environmental conditions in textile workshops, combined with the cylindrical shape and repetitive textural features of yarn bobbins, limit the application of traditional visual solutions. Therefore, we propose a visual measurement method based on the geometric characteristics of binocular imaging: First, all contours in the image are extracted, and the distance sequence between the contours and the centroid is extracted. This sequence is then matched with a predefined template to identify the contour information of the yarn bobbin. Additionally, four equations for the tangent line from the camera optical center to the edge points of the yarn bobbin contour are established, and the angle bisectors of each pair of tangents are found. By solving the system of equations for these two angle bisectors, their intersection point is determined, giving the radius of the yarn bobbin. This method overcomes the limitations of monocular vision systems, which lack depth information and suffer from size measurement errors due to the insufficient repeat positioning accuracy when patrolling back and forth. Next, to address the self-occlusion issues and matching difficulties during binocular system measurements caused by the yarn bobbin surface’s repetitive texture, an imaging model is established based on the yarn bobbin’s cylindrical characteristics. This avoids pixel-by-pixel matching in binocular vision and enables the accurate measurement of the remaining yarn margin. The experimental data show that the measurement method exhibits high precision within the recommended working distance range, with an average error of only 0.68 mm.

## 1. Introduction

In recent years, the degree of equipment automation in Chinese textile workshops has significantly increased. Automated equipment not only simplifies the originally complex mechanical structures but also enables the production of more diverse products, with a notable improvement in stability observed [1,2,3]. However, in this highly automated production process, the detection of yarn margins still relies on manual labor, contrasting sharply with the overall efficiency of the automated production system. To meet the high-precision requirements of modern production scheduling and achieve seamless integration from raw materials to finished products, the accurate detection of yarn margins has become particularly important. The traditional approach involves inspection workers visually observing the remaining yarn on spindles within the workshop and estimating the remaining usage time based on personal experience, manually deciding when to replace the spindles. This method lacks real-time data support, leading to inflexible and unpredictable production planning. Given rising labor costs and limited human resources, this traditional work mode no longer meets the demands of modern production for precise scheduling [4,5,6].

**Figure 1:**
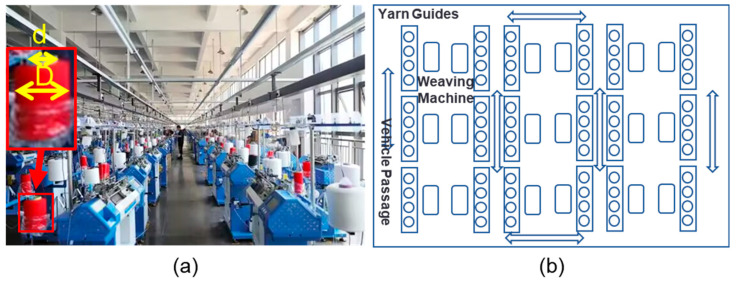
The distribution of weaving machines in a textile workshop: (**a**) a real textile workshop; (**b**) the production layout of the textile workshop.

In these textile workshops, each row contains 10–20 weaving machines, with 5–10 columns. Each weaving machine has an independent yarn supply frame, and each frame vertically holds 1–4 yarn bobbins. The yarn bobbins are connected to the weaving machines via yarn guides (as shown in Figure 1a), and the margin of the yarn is calculated based on the difference in diameter between the yarn bobbin (the full part of the yarn bobbin) and the core (the empty center part of the bobbin). Inspection workers patrol the aisle (in the direction indicated by the arrows in Figure 1b), visually inspecting the remaining yarn on the yarn bobbins. They typically estimate the remaining yarn by comparing the radius difference between the yarn bobbin and the central yarn spindle to decide whether to replace the package. Due to the lack of automated detection methods, workers must frequently move between the aisles, checking the status of each yarn frame individually.

Achieving the automated detection of yarn margins faces several challenges: First, the workshop environment is complex and variable, with yarn bobbins varying in color and size. This variation makes it extremely difficult to extract the contours of the yarn bobbins and position them using traditional threshold segmentation or color analysis methods. Second, the large number of yarn bobbins and their widespread distribution within the workshop make it challenging to efficiently collect image data for all of them. Additionally, the top space structure of the weaving machines is complex, featuring numerous yarn guides, power bridges, and fire protection pipes. These obstacles undermine capturing images from the end faces of the yarn bobbins, further increasing the difficulty of image acquisition.

Current methods primarily rely on tension and weight sensors to detect whether the yarn has been depleted; however, these methods only provide information on whether depletion has occurred and do not enable the real-time monitoring of the remaining yarn quantity [7,8,9]. To improve the accuracy and real-time capability of monitoring, Zhiwei Shi et al. proposed a monocular vision-based solution [10,11]. This method estimates the remaining amount of yarn by calculating the size of the yarn bobbin from detected contour circles in images of the end face of the bobbin. However, monocular vision systems cannot perceive depth information, which restricts measurement accuracy [12]. Furthermore, the applicability of this approach is affected by obstacles surrounding the bobbin end face. In contrast, binocular vision technology can be used to calculate depth information via pixel-wise matching on acquired images [13,14,15], enabling more accurate comparisons of depth differences between the yarn and the bobbin, thus estimating the remaining yarn quantity. However, in the specific application of bobbin residual detection, self-occlusion issues caused by the cylindrical shape of the bobbin lead to errors in feature point matching within binocular vision systems, especially when parts of the bobbin are occluded by other sections, affecting the accuracy of feature point matching [16,17,18,19,20]. Moreover, the presence of repetitive texture features on the surface of the bobbin increases the complexity of feature point matching, so traditional binocular vision algorithms struggle to distinguish similar areas, further impacting the accuracy and reliability of bobbin size measurements.

To overcome existing detection methods’ limitations, we propose a visual measurement method based on the geometric characteristics of binocular imaging. This method constructs an imaging model between the binocular camera and the bobbin, extracting dimensional information directly from it. Compared with existing methods, this approach not only provides binocular depth perception but also addresses inaccurate feature point matching due to self-occlusion and repetitive textures. Additionally, extracting dimensional information directly from the imaging model avoids the need for the pixel-wise matching computation step required in traditional binocular vision systems, enhancing the computational efficiency. This method not only offers the textile industry a more precise and efficient solution for monitoring the remaining yarn quantity but also provides new insights into the dimensional measurement of objects with similar geometric characteristics.

The rest of this paper is organized as follows: Section 2 provides an analysis of the current research status. Section 3 introduces the proposed binocular imaging model for yarn bobbins and its measurement principles. Section 4 details the experimental procedures and measurement results and Section 5 summarizes this study.

## 2. Related Works

Currently, few attempts are being made in the industry to solve similar problems. Common methods involve using tension sensors or weight sensors to detect changes in tension to determine when the yarn bobbin is depleted [7,8,9]. However, these methods have significant limitations, as they only detect whether the yarn is exhausted and cannot provide real-time monitoring or sufficient precision. In contrast, vision-based detection methods offer higher flexibility and accuracy [10,11,12,13,14,15]. Among these methods, monocular vision systems typically require only one camera, making them relatively inexpensive in terms of hardware, easy to deploy and maintain, and simple to install and set up without the need for complex calibration procedures. They can quickly acquire and process images, leading to their widespread use in the textile industry. On the other hand, binocular vision technology has shown great potential in recent years as it non-invasively provides depth information about the measured object, enabling precise perception in three-dimensional space. In the textile industry, the application of binocular vision technology is gradually expanding to areas such as fiber identification and fabric defect detection. However, the real-time and accurate detection of yarn margins using binocular vision is still in the exploratory stage. The main research content of vision-based detection methods includes the topics outlined below.

### 2.1. Monocular Solution

Zhiwei Shi, Weimin Shi, and Junru Wang proposed a low-cost and high-precision method for detecting the edges of yarn bobbins based on monocular vision [10]. Their method uses a monocular camera mounted on a mobile robot to capture images and calculate the edges of the yarn bobbins using monocular vision techniques. Additionally, they introduced an improved neural network algorithm [11]. This method integrates an enhanced YOLO algorithm with contour detection algorithms. The YOLO model detects each yarn bobbin and its size, and based on the detection results from YOLO, the contours and dimensions of each bobbin are then accurately extracted. The diameters of the yarn bobbins detected by both the YOLO and contour detection algorithms are fused, and the lengths and edges of the bobbins are calculated as measurement values. To completely eliminate error detection, the method estimates the remaining yarn amount using the yarn consumption rate and fuses the measurement values with the estimated values using a Kalman filter, enabling the real-time detection of yarn margins.

### 2.2. Binocular Solution

Some methods using binocular vision have been developed, primarily for optimizing surface reconstruction. For example, Wang M, Sun Q, Gao C et al. proposed a 3D visual measurement method based combining dual-line structured light [13]. This method allows the scanning laser plane to slide along a rail while intersecting with the positioning laser plane, eliminating the need to determine the scanning direction and step size. Zhou Y, Zhao J, and Luo C developed a new method for reconstructing general 3D curves from stereo images [14]. This method uses B-spline curve fitting techniques to fit the 2D edge point sets extracted from stereo images. It then constructs conic surfaces using the approximated parametric curves and finds the corresponding conic surfaces’ intersections using a robust iterative algorithm to recover the 3D curves. This method can reconstruct both open and closed 3D curves, meeting the precision requirements of various practical applications. Li G and Zucker S W proposed a method for extending stereo vision to surfaces using differential geometric consistency [15]. This method involves differential geometric studies of surfaces and supports the use of Cartan’s moving frame model on locally quadratic approximations of smooth surfaces to encode geometric context information. This ensures geometric consistency in depth and surface normal. The authors demonstrated the importance of using this geometric context information through a simple stereo algorithm and showcased its powerful detection capabilities on facial images.

Monocular vision detection methods are widely used in various applications due to their low cost and ease of implementation. However, monocular vision systems lack depth information and require a fixed working distance to ensure measurement accuracy. Before use, the system must be calibrated to obtain the relationship between pixels and actual physical dimensions at the specified working distance. During inspection tasks, the system’s limited repeatability accuracy can cause changes in the shooting position, leading to variations in the working distance. To maintain measurement accuracy, recalibration is necessary. Although binocular vision technology can non-invasively provide depth information about the measured object, enabling precise perception in three-dimensional space, it has limitations when applied to yarn margin detection. The primary issues arise from the cylindrical shapes of the yarn packages, which cause self-occlusion, and the repetitive texture features on the surface of the yarn packages. Self-occlusion leads to errors in feature point matching in binocular vision systems, especially when parts of the yarn package are obscured by other parts, resulting in inaccurate feature point matching. Additionally, the repetitive texture features on the surfaces of the yarn packages increase the difficulty of performing feature point matching, making it challenging for traditional binocular vision algorithms to distinguish similar texture regions, which further affects the accuracy of 3D reconstruction. Therefore, traditional binocular vision methods struggle to achieve high-precision real-time detection for objects with special geometric shapes and texture features such as yarn packages. This paper proposes an improved binocular vision method to overcome these limitations.

## 3. Methods

### 3.1. Measurement Principle

**Figure 2:**
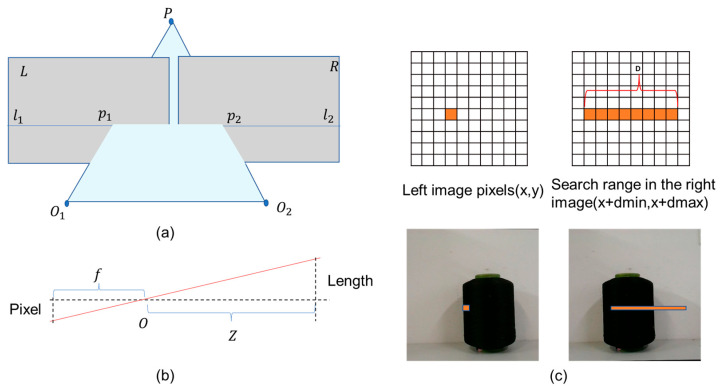
A schematic diagram of binocular stereovision measurement: (**a**) the principle of binocular triangulation. Here, P is a point in the world coordinate system, $p_{1}$ and $p_{2}$ are the image points on the image planes L and R, and $l_{1}$ and $l_{2}$ are the epipolars. (**b**) A basic model of a pinhole camera. The length in world coordinates is imaged as the pixel on the imaging plane through the camera’s optical center O, f is the camera focal length, and Z is the distance between point and the binocular camera. (**c**) The process of binocular pixel matching.

The basic principle of conventional binocular stereovision for depth measurement is shown in Figure 2a. Point P in 3D space could be determined based on the intersection of the lines connecting the projection centers of the two cameras with the imaging points $p_{1}$ and $p_{2}$ [21]. Using the principle of similar triangles, the distance Z from point P to the camera could be calculated. After the depth information was obtained by matching pixels one by one according to the process shown in Figure 2c, the relationship between pixel length and real length could be calculated using the pinhole camera model shown in Figure 2b, thereby calculating the planar dimensions.

When using conventional binocular schemes to perform stereo reconstruction of yarn bobbins, the following issues arise: 1. Matching Failure Due to Self-Occlusion—In the right camera view, parts visible in the left camera view may not be seen, appearing as missing or invisible regions in the right image. This self-occlusion prevents the binocular matching algorithm from finding corresponding pixel points in these regions, leading to matching failures in the occluded areas. This results in the loss of depth information for these parts of the cylinder, affecting the reconstruction of the entire scene’s depth map [11,12]. 2. Impact of Repeated Textures on the Surface of the Yarn Bobbin—The surface of the yarn bobbin has a large number of repeated textures, which makes traditional feature-based matching algorithms less effective. The repeated textures make it difficult to distinguish similar regions during the matching process, thus affecting the accuracy and stability of matching [16,17]. 3. Effect of the Pinhole Camera Model on Cylindrical Objects—Due to the pinhole camera model, the width of the cylinder captured in the image does not directly represent the cylinder’s actual diameter. This means that the width in the image does not directly reflect the cylinder’s actual diameter. This distortion affects the measurement of the cylinder’s diameter based on the image.

**Figure 3:**
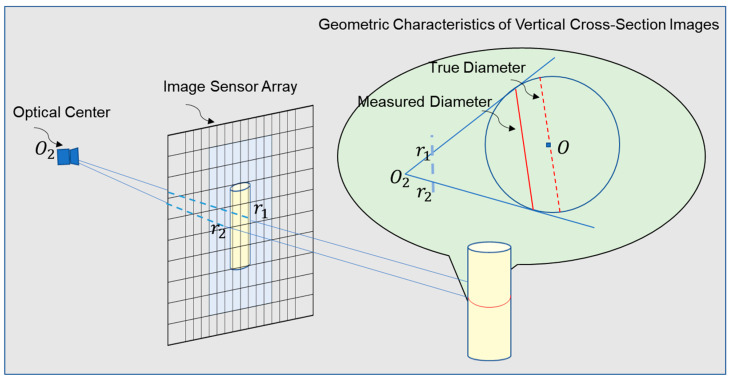
The monocular camera imaging process, where light rays pass through the cylinder, cross the image plane at points $r_{1}$ and $r_{2}$, and converge at the optical center $O_{2}$. Point O represents the center of the cylinder’s circular cross-section.

To address the aforementioned issues, we proposed a visual measurement method based on the geometric characteristics of binocular imaging, considering the imaging features of yarn bobbins in binocular cameras. First, we observed the imaging process of a single camera, as shown in Figure 3: The yarn bobbin was considered to be a cylinder with its axis perpendicular to the ground. We focused on a cross-section of the cylinder perpendicular to its axis, which was regarded as a circular plane. When light passed through the cylinder and entered the camera lens, it formed a series of pixel points on the camera’s imaging plane. These pixel points constituted the projection of the cylinder on the imaging plane. For a cross-section of the cylinder along a vertical axis, we obtained a segment of pixel points on the camera’s imaging plane, with the endpoints being the projections of the outer contour points. According to the pinhole camera model, the projection points of the outer contour on the imaging plane did not correspond to the outermost edge points of the cylinder’s cross-section. Instead, they corresponded to the imaging points of the two tangent lines that passed through the camera’s optical center and were tangent to the circular cross-section of the cylinder. As shown in Figure 3, the red dashed line represents the true diameter of the circular cross-section, whereas the projection size corresponding to the outer contour projection points on the image plane is indicated by the red solid line. Estimating the diameter or width of the cylinder using the outer contour projection points introduced errors, making the calculation inaccurate. This error was related to the shooting distance and the cylinder’s diameter, and it was difficult to decouple. Therefore, the outer contour projection points in monocular vision could not be used to estimate the diameter or width of the cylinder, and additional information was required. Thus, we considered a binocular solution.

**Figure 4:**
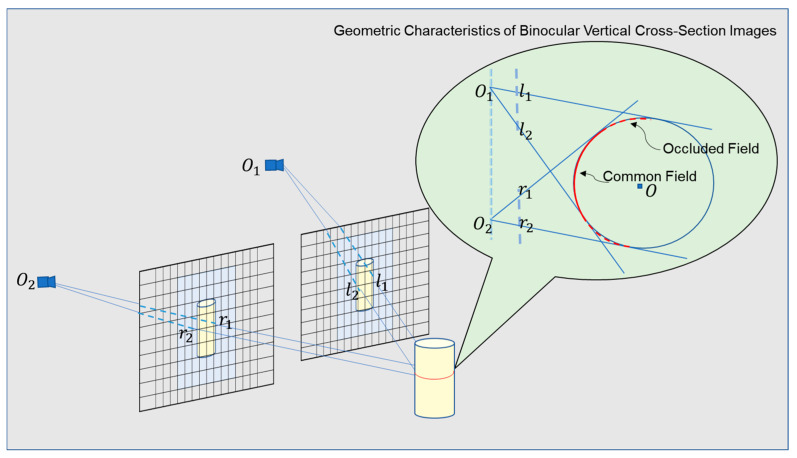
Binocular camera imaging process.

The imaging process of a binocular camera system is illustrated in Figure 4. In this system, light rays converge from different angles at the optical centers $O_{1}$ and $O_{2}$, forming corresponding pixel points on the image planes. Due to the cylindrical shape of the yarn bobbin, certain parts may be occluded by other sections, resulting in one camera being unable to see feature points visible to the other camera; this phenomenon is referred to as self-occlusion. In the figure, the self-occlusion area is marked with a red dashed line, while the region visible to both cameras is indicated by a red solid line. Self-occlusion in binocular vision can lead to inaccurate feature point matching, thereby undermining the calculation of depth information and the measurement of the remaining yarn quantity.

**Figure 5:**
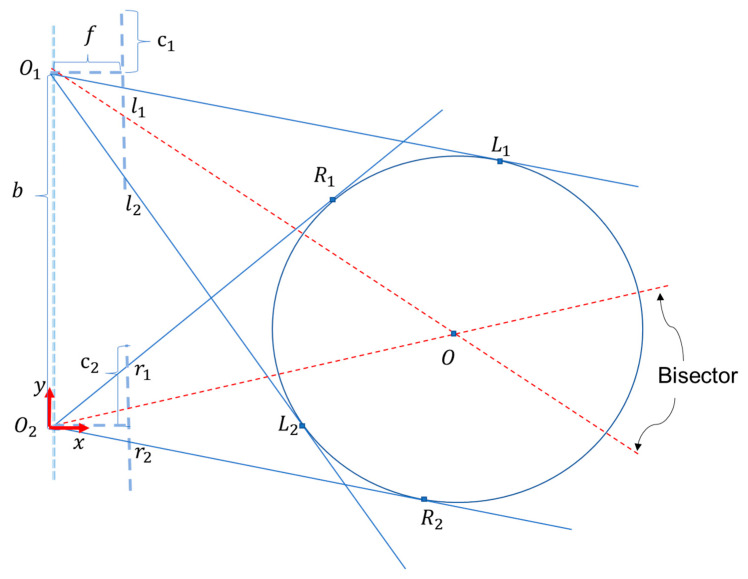
The imaging process of the cross-section of the yarn bobbin along a vertical axis. Here, $c_{1}$ and $c_{2}$ are the horizontal coordinates of the camera optical centers on the pixel plane; the outer contour points $l_{1}$, $l_{2}$, $r_{1}$, and $\;r_{2}$ are the real-space points $L_{1}$, $L_{2}$, $R_{1}$, and $\;R_{2}$; f is the camera focal length; and b is the baseline of the binocular camera.

Further, the imaging process for the circular cross-section in the binocular camera is shown in Figure 5. In the imaging plane of the left camera, points $L_{1}$ and $L_{2}$ on the circle correspond to the outer contour points $l_{1}$ and $l_{2}$. The two lines $\;{line}_{O1-l1}$ and ${line}_{O1-l2}$, passing through the outer contour points and converging at the camera’s optical center, are tangent to the circle. In the imaging plane of the right camera, points $R_{1}$ and $R_{2}$ on the circle correspond to the outer contour points $r_{1}$ and $r_{2}$. The two lines ${line}_{O2-r1}$ and ${line}_{O2-r2}$, passing through the outer contour points and converging at the camera’s optical center, are also tangent to the circle. For a calibrated and corrected binocular system, the coordinates of the camera optical centers, the coordinates of the outer contour points on the imaging planes, the focal lengths, and the baseline distance are all known parameters. Based on these parameters, the equations of the light rays converging at the optical centers can be obtained. The four light rays could determine a unique circle tangent to them, which corresponded to the outer contour of the yarn bobbin in 3D space. This allowed for the further calculation of the diameter of the yarn bobbin.

More specifically, taking the optical center of the right camera $O_{2}$ as the origin of the coordinate system, the line connecting the optical centers of the two cameras $O_{1}$–$O_{2}$ as the *y*-axis, and the direction perpendicular to $O_{1}$–$O_{2}$ as the *x*-axis, we established a plane coordinate system. In this coordinate system, the coordinates of these points were represented as $O_{1}\left(0,b\right),\;O_{2}\left(0,\;0\right),\;l_{1}\left(f,b-x_{l1}+c_{1}\right),{\;l}_{2}\left(f,b-x_{l2}+c_{2}\right),\;r_{1}\left(f,c_{2}-x_{r1}\right),\;and{\;r}_{2}(f,c_{2}-x_{r2})$. Each outer contour point and its corresponding camera optical center represented a straight line. The equations of the established line were represented as follows:

(1) $$\begin{array}{c} {line}_{o1-l1}:\frac{y-b}{-\left(x_{l1}-c_{1}\right)}=\frac{x}{f} \end{array}$$

(2) $$\begin{array}{c} {line}_{o1-l2}:\frac{y-b}{-\left(x_{l2}-c_{1}\right)}=\frac{x}{f} \end{array}$$

(3) $$\begin{array}{c} {line}_{o2-r1}:\frac{y}{-\left(x_{r1}-c_{2}\right)}=\frac{x}{f} \end{array}$$

(4) $$\begin{array}{c} {line}_{o2-r2}:\frac{y}{-\left(x_{r2}-c_{2}\right)}=\frac{x}{f} \end{array}$$

where $c_{1}$ and $c_{2}$ are the horizontal coordinates of the camera optical centers on the pixel plane; $x_{l1}$, $x_{l2}$, $x_{r1}$, and $\;x_{r2}$ are the horizontal pixel coordinates of the outer contour points; f is the camera focal length; and b is the baseline of the binocular camera.

Given that the four lines are tangents to the circle, we had four independent constraints. By calculating the distance from each line to the assumed circle center and ensuring that these distances equal the radius of the circle, we were essentially looking for a point (the circle center) for which the distances to each of the four lines are the same. Since each line provided information about the position of the circle center, four such conditions were sufficient to determine a unique circle center and its corresponding radius. Therefore, by solving these four distance equations, we found a unique circle center coordinate and a radius that satisfied all the conditions, thus establishing a unique circle. We assumed an arbitrary circle center position (h,k) and radius $r_{0}$. For each line, we used the point-to-line distance formula to calculate the distance from the circle center to the tangent line; this distance had to equal the radius:

(5) $$\begin{array}{c} r_{0}=\frac{\left|mh-k+c\right|}{\sqrt{1+m^{2}}} \end{array}$$

where m is the slope of the line and c is the intercept of the line. By setting up an error function as the difference between the square of the distance from the circle center to the four lines and the square of the radius, we used an iterative optimization method to minimize the error function, thereby determining the circle center and radius:

(6) $$\begin{array}{c} E\left(h,k,r\right)=\sum_{i=1}^{4}\left(\frac{{(m}_{i}h-k+c_{i}{)}^{2}}{1+m_{i}^{2}}-r^{2}\right) \end{array}$$

However, a major drawback of numerical methods is their sensitivity to initial values and tendency to become trapped in local optima. Thus, if the initial estimates are not appropriate, the results may deviate significantly from the true circle center position. Additionally, since the iterative process involves a large amount of computation, this method can be time-consuming in practical applications. We introduced a geometric method to address these issues.

The geometric method involved using the angle bisectors of two intersecting lines within the same imaging plane that intersected at the optical center to determine the circle center position. This method directly determined the circle center using geometric relationships, avoiding complex iterative calculations. First, in the left camera’s imaging plane, we identified two intersecting ${line}_{O1-l1}$ and ${line}_{O1-l2}$ that intersected at the optical center. Similarly, in the right camera’s imaging plane, we identified two intersecting lines ${line}_{O1-r1}$ and ${line}_{O1-r2}$ that intersected at the optical center. Since these four lines were all tangent to the circle, the intersection of their angle bisectors passed through the circle center. Solving for the intersection coordinates of the angle bisectors gave us the circle center coordinates. Calculating the distance from the circle center to any of the tangent lines yielded the radius of the circle.

Since this method used the coordinates of the camera optical centers and the coordinates of the outer contour points on the image to establish equations, its accuracy was limited by two factors: the camera calibration parameters and the precision of the contour coordinate extraction. After calibration and correction, the error introduced by the camera calibration parameters was fixed. This error mainly stemmed from systematic errors during the calibration process, such as inaccuracies in the placement of the calibration board and minor deviations in the camera’s internal parameters. These fixed errors needed to be carefully calibrated to minimize their impact. Secondly, the precision of contour coordinate extraction was the primary factor causing variations in the measurement results. The precision of contour coordinate extraction was closely related to the distance between the camera and the object. When the camera was closer to the object, the details in the image were more pronounced, and the precision of contour point extraction was higher. Conversely, when the camera was further from the object, the image resolution decreased, making it more difficult to extract contour points, and the precision also decreased. In this context, we used the following formula:

(7) $$\begin{array}{c} \hat{e}=\frac{Z*pixel}{f} \end{array}$$

where Z is the distance from the object to the camera, pixel is the pixel size in the image, and f is the camera focal length. This formula indicates that as Z increases, the error $\hat{e}$ also increases.

### 3.2. Contour Localization

In this method, precise contour localization was particularly important because it directly affects the accuracy of geometric feature extraction and dimensional measurements. By accurately locating the contour of each yarn bobbin, we could ensure that the outer contour points of the yarn bobbins were correctly matched during binocular imaging, thereby avoiding errors caused by self-occlusion and repeated textures. In an image containing multiple yarn bobbins, each located in different regions, it was necessary to segment the image into multiple sub-regions, with each sub-region corresponding to an individual yarn bobbin. The ranges of these sub-regions were known.

**Figure 6:**
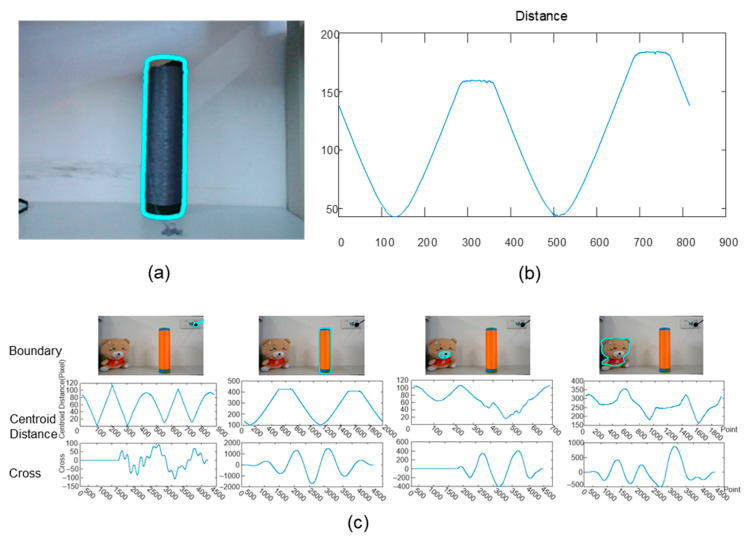
The process of locating the contour of the yarn bobbin using centroid distance: (**a**) the centroid distance sequence template of the yarn bobbin contour; (**b**) the centroid distance sequence of the yarn bobbin contour; (**c**) a schematic of the matching process, where the first row shows the extracted contours, the second row shows the corresponding centroid distance sequences, and the third row shows the results of the cross-correlation function between the extracted centroid distance sequences and the centroid distance sequence template.

After obtaining the sub-region range for each yarn bobbin, the contour of the yarn bobbin within the sub-region had to be localized. In the 2D image projection, the yarn bobbin appears as a characteristic curved polygon shape (Figure 6a), with smooth curves at the top and bottom boundaries and no obvious corners; the side boundaries are straight lines with no significant curvature. This unique geometric structure results in four distinct peaks in the sequence of distances from the centroid of the contour to the edges (Figure 6b). The peaks at the top and bottom boundaries are relatively gentle and broadly distributed, reflecting the boundaries’ smooth curve characteristics; the peaks at the side boundaries are more concentrated and sharper, indicating that they are straight segments. By analyzing the distribution patterns of these peaks, the geometric features of the yarn bobbin could be effectively extracted, providing an important reference point for subsequent measurements and localization.

To achieve the precise localization of the yarn bobbin in the image, we first created a centroid distance sequence template (as shown in Figure 6b), which reflected the typical characteristics of the yarn bobbin contour, namely the four distinct peak distributions. By performing correlation analysis between the centroid distance sequences of the contours in the image to be detected and the template sequence (as shown in Figure 6c), we could evaluate the similarity and matching degree of each contour to the template contour. By calculating the cross-correlation coefficient, we could identify the most matching contour. The contour groups that produced the highest correlation peaks with the template sequence were identified as successfully localized instances. Through these steps, the contour localization of the yarn bobbin could be effectively completed, providing reliable data for subsequent dimensional measurements. This not only helped to improve the accuracy of the measurements but also ensured stable performance under different lighting conditions and background complexities.

## 4. Experiment

First, we performed binocular calibration and epipolar rectification to ensure the accuracy of subsequent measurements. Through single-camera calibration, we obtained the intrinsic and extrinsic parameters of the camera, including the focal length, principal point coordinates, and distortion coefficients, establishing the transformation relationship from the real-world coordinate system to the image pixel coordinates. Using the distortion model, we corrected the distortion phenomena in the images, improving image quality. Binocular calibration further determined the relative positional relationship between the two cameras, addressing issues caused by installation errors. To simplify the pixel matching task, we adopted epipolar rectification technology, transforming the matching problem into a one-dimensional search, significantly reducing computational complexity. Next, we proposed a method based on contour centroid distance to precisely locate the contour of the yarn bobbin. This method involved extracting the distance sequence between the contour and the centroid and matching it with a predefined template to identify the contour information of the yarn bobbin. Finally, we used the angle bisector method to solve for the circle center coordinates. By determining the four tangent lines from the camera optical centers to the contour points and finding the angle bisectors of each pair of tangent lines, we solved the system of equations of these two angle bisectors to find their intersection point, the center coordinate of the outer contour circle of the yarn bobbin in 3D space.

This experimental setup used a binocular camera model HBVCAM-4M2214HD-2 module (the camera was provided by Shenzhen Huibo Vision Technology Co., Ltd., Shenzhen, Guangdong Province, China), which indicates that the camera name is HBVCAM, with a maximum resolution of 4 million pixels and an individual pixel size of 2 μm. It is a binocular camera module that supports high-definition shooting. The camera’s baseline is fixed at 60 mm, and the focal length is 3 mm. In the experiment, the camera’s capture resolution was set to 3840 × 1080, with a frame rate of 30 FPS. The experiment was conducted in both a controlled laboratory environment and a real production workshop. To simulate actual production conditions, natural lighting environments were chosen; both the workshop and the laboratory used standard fluorescent lighting to avoid any influence from special light sources. Additionally, we ensured that the yarn bobbins were in tight contact with the holder to minimize potential errors in experimental results caused by bobbin tilting. The specific steps followed are defined in the subsections below.

### 4.1. Binocular Calibration and Epipolar Rectification

To establish the binocular imaging model for the yarn bobbin, we need to understand the relative positional relationship between the two cameras and the relative positional relationship between the cameras and the yarn bobbin. Therefore, binocular calibration [22] is required for the cameras. The binocular calibration used process is as follows:

We perform single-camera calibration for both cameras to obtain parameters such as focal length, principal point coordinates, and distortion coefficients, thereby establishing the transformation relationship from real-world coordinates to image pixel coordinates. Points in the real world are transformed from the world coordinate system $O_{w}$ to the camera coordinate system $O_{c}$. The camera coordinate system $O_{c}$ uses the camera’s optical center as the origin and the camera’s optical axis as the *z*-axis. This transformation is a rigid transformation, obtained through rotation R and translation T.

(8) 1Zcuv1=1dx0u001dyv0001f0000f000001RT01xwywzw1

In Equation (8), $x_{w},\;\;y_{w},\;{\;z}_{w}$ are the coordinates of a point in the world coordinate system $O_{w}$, R is a 3 × 3 rotation matrix, and T is a 3 × 1 translation matrix. Then, after perspective transformation from the camera coordinate system $O_{c}$ to the image coordinate system $O_{uv}$, f is the focal length, x and y are the coordinates in the image coordinate system $O_{uv}$, and $Z_{c}$ is the scale factor. Finally, since the actual image sensor is a grid of pixels, the continuous image coordinates in the image coordinate system $O_{uv}$ are converted into discrete pixel coordinates u,v in the pixel coordinate system $O_{u_{0}v_{0}}$. $d_{x}$ and $d_{y}$ represent the physical dimensions of each pixel in the pixel coordinate system along the *x*-axis and *y*-axis, respectively.$\;u_{0}$ and $\;v_{0}$ are the coordinates of the camera optical center in the pixel coordinate system $O_{u_{0}v_{0}}$.

Using only a linear model is insufficient to overcome the negative effects caused by inherent lens defects, so a distortion model is introduced to correct the distortion of the microscope. The complete distortion model used in this paper is as follows:

(9) $$\begin{array}{c} \left\{\begin{array}{c} x^{\prime }=x\left(1+k_{1}r^{2}+k_{2}r^{4}\right)+p_{1}\left(r^{2}+2x^{2}\right)+2p_{2}xy\\ y^{\prime }=y\left(1+k_{1}r^{2}+k_{2}r^{4}\right)+p_{2}\left(r^{2}+2y^{2}\right)+2p_{1}xy \end{array}\right. \end{array}$$

where $\left(x^{\prime },y^{\prime }\right)$ are the normalized pixel coordinates after distortion, (x,y) are the ideal undistorted normalized pixel coordinates, r is the distance from the pixel point to the image center, and $r^{2}=x^{2}+y^{2}$. $\left(k_{1},\;k_{2}\right)$ are the radial distortion coefficients, whereas $\left(p_{1},\;p_{2}\right)$ are the tangential distortion coefficients.

Equations (8) and (9) establish the imaging model that maps 3D points in the real world to pixel positions on a 2D image, using Zhang Zhengyou’s calibration method, based on a chessboard pattern, to obtain camera parameters.

**Figure 7:**
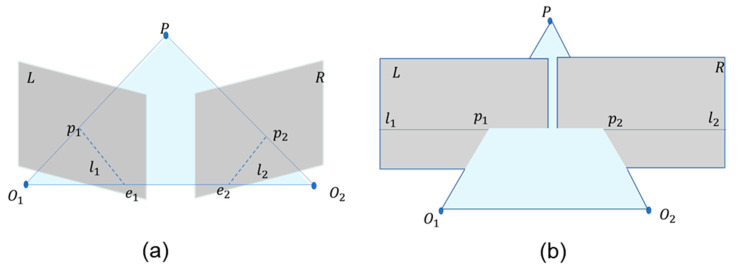
A schematic of epipolar geometry, where P is a point in the world coordinate system, $p_{1}$ and $p_{2}$ are the image points on the image planes L and R, and the epipoles $e_{1}$ and $e_{2}$ are the intersections of the baseline $O_{1}O_{2}$ with the image planes L and R. At this point, the plane formed by $O_{1}O_{2}P$ is called the epipolar plane: (**a**) the original epipolar geometry diagram; (**b**) the epipolar geometry diagram after epipolar rectification.

Pixel matching is a crucial step in binocular vision, and it involves finding corresponding pixel points in two images. The projection positions of the same world point in the left and right images differ due to the viewing angle differences. By measuring the disparity, the depth information of points in the scene can be inferred according to the principle of triangulation. To reduce the computational load, additional constraints must be introduced. After calibrating to obtain the intrinsic and extrinsic parameters of both cameras, the two cameras’ relative positions in a binocular system, due to installation and manufacturing errors, are not in the same plane but have a certain angle. Their imaging satisfies the epipolar geometry constraint shown in Figure 7a [23].

Based on the principles of epipolar geometry, the pixel point $p_{1}$ in the left view can be mapped to the epipolar $l_{2}$ in the right view, significantly narrowing the search range for its corresponding homonymous point $p_{2}$. However, calculating the epipolar for each pixel individually still requires significant computation. In fact, the associated epipolars $l_{1}$ and $l_{2}$ lie in the same epipolar plane and share an epipolar constraint. As the epipolar plane rotates, all image pixels can be represented by some epipolar. By reordering the pixels on the epipolar, pixels on the same epipolar are aligned in the same row, allowing the pixel point $p_{1}$ in the left view to be directly associated with its homonymous candidate pixels in the right view using the row number. These candidate pixels differ only in terms of their column coordinates. This process, known as epipolar rectification (as shown in the process from Figure 7a,b), essentially transforms the binocular vision system into an ideal configuration where the imaging planes of the two cameras are coplanar and vertically aligned, simplifying pixel matching.

### 4.2. Yarn Bobbin Contour Coordinate Extraction

**Figure 8:**
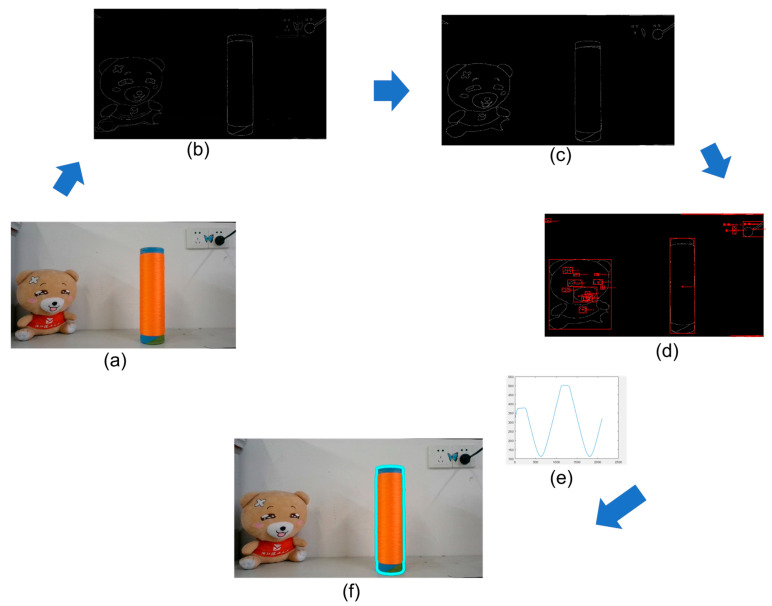
The contour localization process: (**a**) original image; (**b**) contours detected using structured forests; (**c**) contours after filtering; (**d**) extracted contour centroids; (**e**) centroid distance sequence image; (**f**) image after the localization of yarn bobbin contours.

After camera calibration and epipolar rectification, the next step is to extract the cylindrical yarn bobbin coordinates in the captured images. This process requires locating the yarn bobbin coordinates in the epipolar-rectified images. We propose a method that combines structured forest edge detection with the centroid distance method to achieve the precise localization of the yarn bobbins in the images. This method is based on the correlation analysis between the contour centroid distance sequence and a predefined template sequence. The steps are as follows:

- Perform edge contour detection on the image based on structured forests [24,25,26], as shown in Figure 8b.
- Filter out low-threshold contours and extract the centroids of the remaining contours; the filtering result is shown in Figure 8c, and the extracted centroids are shown in Figure 8d.
- Conduct correlation analysis between the distance sequence from the contour to the centroid and the template sequence (Figure 8e). First extract the centroid distance (distance from the centroid to the contour) sequence of a reference yarn bobbin contour as the template (Figure 8e).
- Perform correlation analysis between this template sequence and the centroid distance sequences of all possible yarn bobbin contours in the image to be detected. By calculating the cross-correlation coefficients, evaluate the similarity and matching degree of each contour to the template contour. The results of this process are shown in Figure 8c–f, where each figure represents the correlation analysis results for different contours with the template sequence. The contour groups that produce the highest correlation peaks with the template sequence are identified as successfully localized instances.
- Select the contour with the maximum correlation coefficient as the final result; the localization result is shown in Figure 8f.

### 4.3. Results

Using the parameters of the binocular camera system obtained through calibration (including the coordinates of the camera optical centers, the focal length, the baseline distance, and the coordinates of the contour points on the imaging plane), we first determined the four tangent lines from the camera optical centers to the contour points. Then, we found the angle bisectors of each pair of tangent lines (two tangent lines from the left camera and two tangent lines from the right camera) and solved the system of equations of these two angle bisectors to find their intersection point. This intersection point is the center coordinate of the outer contour circle of the yarn bobbin in 3D space. The radius value of the yarn bobbin can be calculated as the distance from the center to any of the tangent lines.

To verify the measurement accuracy of this method at different distances and positions, the experiment detailed below was designed.

#### 4.3.1. Measurement Results at Different Distances

The primary objective of this experiment is to evaluate the measurement accuracy of the camera for the yarn bobbin samples at different distances by gradually adjusting the distance between the camera and the yarn bobbin. This is carried out so that the yarn bobbin occupies a progressively smaller portion of the image, decreasing from the largest possible frame down to smaller sizes. The goal is to determine an optimal working distance range that ensures the accuracy and reliability of the measurement results.

**Figure 9:**
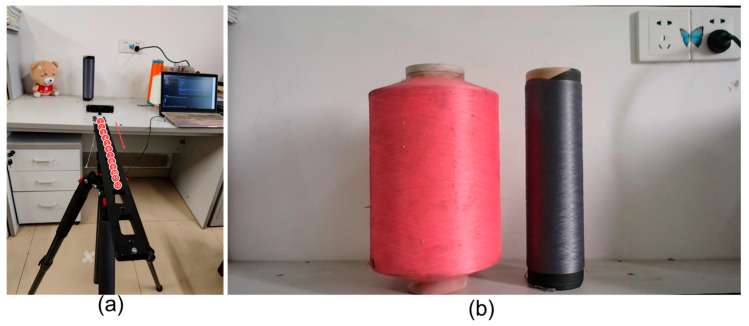
Measurement results at different distances: (**a**) a schematic of measurements at different distances for the yarn bobbin, numbers 1–12 represent the sequential positions on the camera mount, and the arrow indicates the direction of camera movement; (**b**) the measured yarn bobbin samplesfrom left to right: yarn bobbin1; yarn bobbin2.

**Figure 10:**
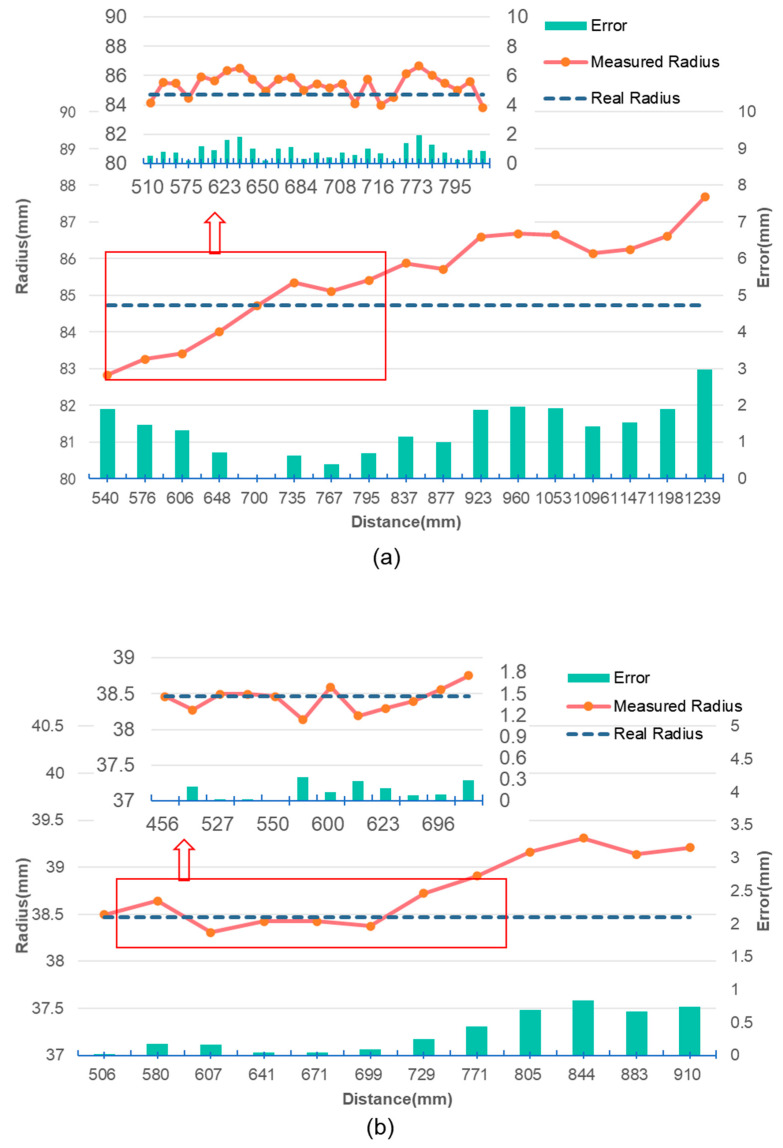
Measurement results at different distances: (**a**) the measurement results of yarn bobbin1 at different distances; (**b**) the measurement results of yarn bobbin2 at different distances.

In the experiment, we selected yarn bobbin samples with the maximum and minimum margins and fixed them in position. The camera was mounted on a tripod. We gradually adjusted the distance between the camera and the yarn bobbin, recording the measurement results at different distances (Figure 9). The measurement results are shown in Figure 10a,b, where the *x*-axis represents the distance between the camera and the yarn bobbin and the *y*-axis represents the measured radius of the yarn bobbin. The solid blue line indicates the actual measured radius of the yarn bobbin, while the dashed red line represents the standard value.

For the sample with the maximum margin (Figure 10a), at closer distances (540 mm to 700 mm), the measurement results are basically consistent with the standard value, with minimal error, which is approximately 0%. As the measurement distance increases, the measurement results start to deviate from the standard value, and the error gradually increases. In particular, when the distance exceeds 700 mm, the measurement results significantly increase, indicating a larger deviation, which aligns with the error model described in Equation (7). Multiple measurements within the working range of 540 mm to 700 mm showed that the measurement values were very close to the standard radius, demonstrating high repeatability and stability.

For the measurement results of the sample with the minimum margin (Figure 10b), the performance is similar to that in Figure 10a, with the optimal working range extending from 505 mm to 671 mm. Within this range, the measurement results are largely consistent with the standard value, with the error remaining at a low level. However, as the size of the yarn bobbin decreases, the optimal working range narrows, indicating that the size of the yarn bobbin significantly affects measurement accuracy. Combining the results from Figure 10a,b, considering that the optimal working range for smaller yarn bobbins is also narrower, the recommended optimal working range in this experimental environment is the intersection of the working ranges for the maximum and minimum margin samples, approximately 540 mm to 671 mm. To ensure the measurement results’ accuracy and reliability, it is recommended to perform measurements within the distance range of 540 mm to 671 mm. Within this range, the measurement results are highly consistent with the standard value, with the error maintained at a low level, effectively avoiding measurement deviations caused by increased distance. This can lead to more precise measurement data in practical applications.

#### 4.3.2. Measurement Results at Different Positions

The main objective of this experiment is to evaluate the camera’s measurement accuracy using yarn bobbin samples at different positions and gradually adjusting the relative position between the camera and the yarn bobbin within the recommended working distance range. Specifically, the yarn bobbin was moved from a position near the right edge of the image to the left edge. This was carried out to assess the camera’s measurement precision at the same distance but at different positions, aiming to determine the optimal working position range and further improve the accuracy of measurement results.

**Figure 11:**
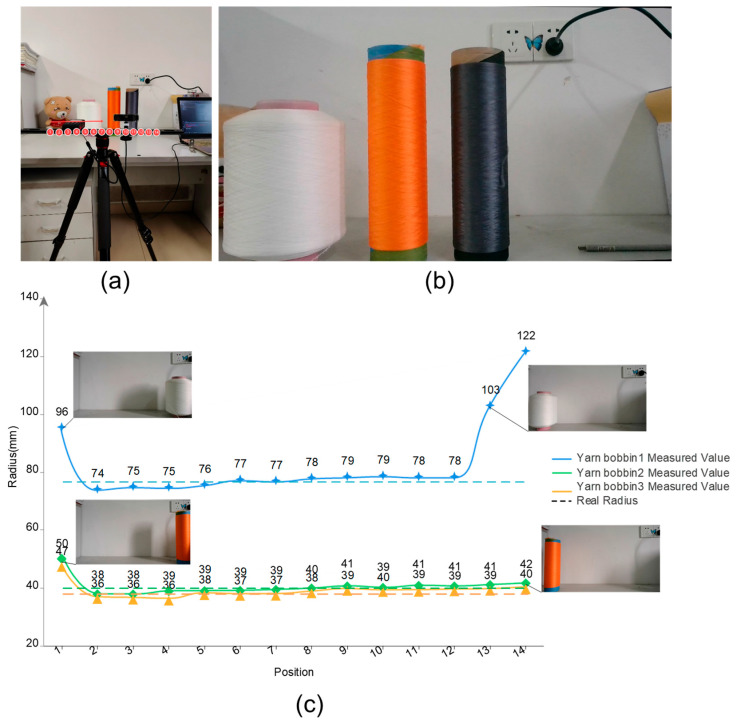
Measurement results at different angles: (**a**) a schematic of measurements at different camera positions, numbers 1–14 represent the sequential positions on the camera mount, and the arrow indicates the direction of camera movement; (**b**) measured yarn bobbin samples—from left to right: yarn bobbin1, yarn bobbin2, and yarn bobbin3; (**c**) measurement sizes at different camera positions.

In the experiment, we analyzed the measurement results of the yarn bobbin samples at different camera positions, as shown in Figure 11c. The results indicate that the most accurate measurements are obtained when the yarn bobbin is located at the center of the image. Horizontal displacement from this central position increases the measurement error. Furthermore, we observe that if the distance between the yarn bobbin’s contour and the image edge is less than 20 pixels, the accuracy of the measurements significantly decreases, increasing the error. This is due to the barrel distortion present during the distortion correction process, which is more pronounced at the edges of the image, causing stretching in the corrected edge regions and blurring at the yarn bobbin edges and thereby reducing the precision of contour extraction. This observation is consistent with our previously mentioned distortion model (Equation (9)). To achieve precise measurement results, it is recommended to place the yarn bobbin at the center of the image and ensure that its contour maintains a distance of at least 20 pixels from the image edges. Through this experiment, we determined the optimal capture range within the images taken by the used camera, minimizing distortion’s impact on measurement accuracy.

#### 4.3.3. Measurement Results from Different Methods

**Figure 12:**
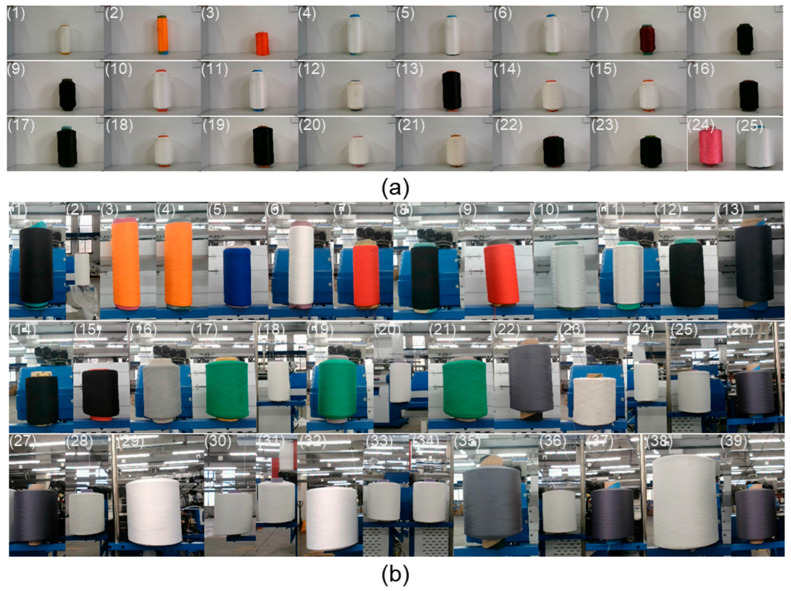
Yarn bobbin sample, the numbers in the figure represent the samples numbered in ascending order based on the size of the yarn bobbin: (**a**) a sample captured in the laboratory; (**b**) a sample captured in the production workshop.

**Figure 13:**
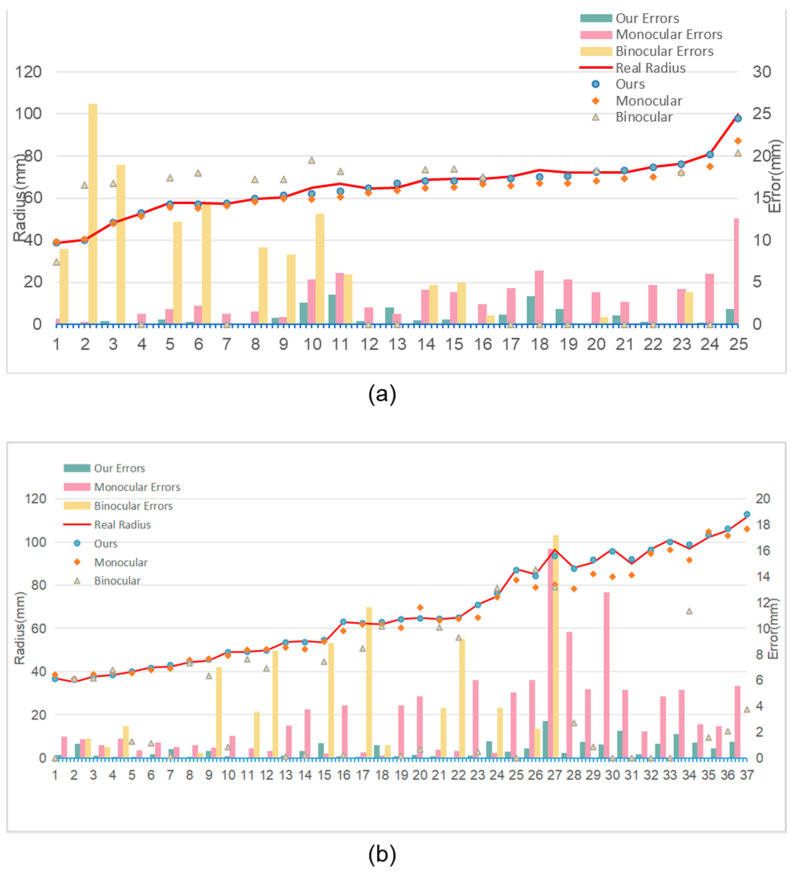
Measurement results. The points in the binocular vision method that did not match successfully resulted in excessively large errors, which are not displayed in the error bar chart in the figure: (**a**) a comparison of measurement errors in yarn bobbin radius using different methods in a laboratory environment; (**b**) a comparison of measurement errors in yarn bobbin radius using different methods in a production workshop environment.

In both the laboratory and the production workshop, the dimensional measurement of yarn bobbin samples was conducted using three different methods: the monocular vision method, the binocular vision method, and the proposed method. The monocular vision method used in the experiment relies on a pre-calibrated relationship between pixels and actual length, determining the size of the yarn bobbin based on the pixel dimensions occupied by its width. The binocular vision method calculates depth information by computing the disparity between images obtained from two different viewpoints using the SGBM algorithm described in [27], thereby calculating the depth difference between the yarn bobbin and the core to determine the bobbin’s size. The samples measured are shown in Figure 12. The measurement results are presented in Table 1 and Figure 13.

In the data in Table 1, we can clearly observe that our proposed measurement method has significant advantages with regard to error control. Across all samples, our method not only maintains a low average error but also exhibits superior stability and consistency compared to the other two methods. Specifically, the average error of our method is approximately 0.68 mm in the laboratory environment and further decreases to 0.65 mm in the production workshop environment. This result fully demonstrates the robustness and reliability of our method across different environments. In contrast, while the monocular vision method ranks second in terms of error control, its performance is constrained by the need to accurately know the distance between the camera and the yarn bobbin beforehand. In practical applications, this necessitates additional measurement steps to determine this distance, undoubtedly increasing operational complexity and inconvenience. Moreover, due to the lack of depth information, the monocular vision method has inherently limited accuracy and cannot match our method’s precision. The binocular vision method, although theoretically capable of providing depth information, exhibits significant fluctuations in error in this study. This is mainly due to self-occlusion and texture matching caused by the cylindrical geometry and surface characteristics of the yarn bobbins. In the binocular vision method, these issues lead to matching failures, including extreme cases where the results are zero, which is unacceptable in practical applications. Additionally, mismatched points introduce substantial errors, severely impacting the measurement results’ accuracy. Conversely, our method addresses these critical issues in binocular vision through optimized imaging models and algorithms. Instead of relying on complex pixel-by-pixel matching processes, our method acquires spatial information by extracting and matching contours. This approach not only enhances computational efficiency but also significantly improves measurement accuracy. Test results in both laboratory and workshop environments show that our method provides measurements closer to the true values (Figure 13).

In Figure 13, we can observe the following: in both the laboratory and production workshop, the monocular vision measurement method (represented by orange diamonds) consistently underestimates the true diameters of the yarn bobbins, a trend particularly evident in samples with larger diameters. This observation aligns with the imaging model discussed in Figure 3.

The binocular vision measurement method (represented by light yellow triangles) exhibits significant fluctuations in error, sometimes resulting in matching failures and zero disparity, as indicated by the omission of some error bars for this method due to excessively large errors. The accuracy of the binocular method, which relies on image matching and depth estimation, is influenced by multiple factors, including image quality and lighting conditions. Errors in depth estimation primarily stem from inaccuracies in matched points and simplifying assumptions made during depth calculation. Mismatches and zero-disparity issues mainly arise from image matching algorithms’ instability when handling the complexity of the yarn bobbin surface.

In contrast, the measurements obtained using our proposed method (represented by blue circles) are highly consistent with the true values (red solid line). In the laboratory environment, the average error of our method is approximately 0.68 mm, while in the production workshop environment, it further decreases to 0.65 mm. By optimizing the imaging model, our method effectively addresses self-occlusion and texture matching issues, providing accurate measurement results across different yarn bobbin sizes. The primary sources of error are mainly due to the precision of contour extraction. Factors such as contour spurs and concave edges can introduce errors of 1–2 pixels in size. According to Equation (7), this translates to an error range of 0–0.89 mm within the recommended measurement range. Additional sources of error include noise, minor inaccuracies in camera calibration, and computational errors during image processing. However, the average error within the recommended range remains below 1 mm, meeting the precision requirements of enterprises and providing a viable inspection solution.

## 5. Discussion

In the specific scenario of residual yarn detection, traditional binocular vision matching algorithms such as AD-CENSUS and SGBM have significant limitations [27]. These algorithms exhibit notable declines in the accuracy and efficiency of feature point matching, especially under poor lighting conditions, when processing objects such as yarn bobbins, which have highly homogeneous surfaces and cylindrical geometric structures. These challenges directly impact the precision of the acquisition of depth information, thereby affecting the reliability of residual detection. To overcome these limitations, this study proposes an improved binocular algorithm specifically optimized for yarn bobbins’ characteristics. Our method does not rely on texture features on the surface of the yarn but focuses on the prominent feature of the bobbin’s contour. By processing epipolar-corrected images to extract and match contours, our method ignores all pixels outside the contour in the image, concentrating on obtaining spatial information from contour pixels. Combined with our imaging model for yarn bobbins, this approach achieves high-precision measurements even under uneven lighting or when surface textures are not distinct. Experimental results show that our method has an average error of less than 1 mm, meeting industrial application requirements and demonstrating its feasibility and effectiveness in actual production environments.

The proposed algorithm significantly reduces computational complexity by focusing on contour extraction and establishing an imaging model based on contour coordinates, enabling the rapid provision of measurement results in production environments without long computational delays. Our method has a time complexity of O(m∗n), notably lower than those of traditional binocular vision methods. For example, using the SGBM algorithm for disparity calculations leads to a time complexity of $O(m^{2}*n^{2}*d)$ or higher (for an m∗n grayscale image, the disparity search range is d), depending on the specific implementation and optimization level of the algorithm. The disparity optimization process, including disparity map smoothing and disparity calculations, also adds additional computational overhead. In contrast, our method exhibits a clear advantage in time efficiency, particularly important for real-time production environments.

Our algorithm is specifically designed for measuring the dimensions of objects with rotary body shapes such as yarn bobbins, but its versatility mainly lies in its adaptability to rotary body shapes. Cylindrical, conical, and other rotary body shapes are very common in industrial products. By analyzing objects’ contour features rather than relying on surface textures or complex image processing techniques, our algorithm can be widely applied to measuring the dimensions of various rotary body-shaped items, including yarn bobbins, pipes, bearings, tires, and other industrial products. Looking forward, there is enormous potential to expand our algorithm’s applicability by incorporating more geometric characteristics. For example, integrating shape recognition technology into the algorithm could enable it to automatically identify and adapt to different shapes. Additionally, introducing machine learning techniques could allow the algorithm to learn and adapt to the specific geometric properties of various solids, thereby improving its measurement accuracy and robustness. Through these optimizations and expansions, our algorithm holds promise for broader applications, bringing innovation and value to other industries.

## Figures and Tables

**Table 1:** The table compares the yarn bobbin radius measurement results obtained using different methods in both laboratory and production workshop environments, with units given in millimeters (mm).

Environment	Yarn Bobbin Number	Monocular		Binocular		Ours		Real Radius
		Value	Error	Value	Error	Value	Error	
laboratory	1	39.20	0.68	29.59	8.93	39.07	0.55	38.52
	2	40.40	0.34	66.29	26.22	40.17	0.11	40.07
	3	48.01	0.03	67.03	18.99	48.39	0.35	48.04
	4	51.57	1.27	0.00	52.84	52.91	0.07	52.84
	…							…
	23	72.04	4.19	72.43	3.80	76.19	0.04	76.23
	24	74.98	5.99	0.00	80.96	80.73	0.23	80.96
	25	87.28	12.61	81.48	18.41	98.04	1.85	99.89
	Average error		3.41		41.13		0.68	
workshop	1	38.57	1.67	0.00	36.90	36.64	0.26	36.90
	2	36.62	1.47	36.65	1.49	36.28	1.12	35.16
	3	38.71	1.01	36.87	0.84	37.51	0.20	37.71
	4	39.88	1.49	40.83	2.44	38.42	0.02	38.39
	…							…
	35	104.74	2.60	9.46	92.69	103.35	1.20	102.15
	36	102.86	2.47	12.38	92.96	106.09	0.76	105.34
	37	105.99	5.56	22.48	89.07	112.80	1.26	111.55
	Average error		3.35		34.42		0.65	

## Data Availability

Data are contained within this article.
